# Determinants of left ventricular ejection fraction and a novel method to improve its assessment of myocardial contractility

**DOI:** 10.1186/s13613-019-0526-7

**Published:** 2019-04-16

**Authors:** Manuel Ignacio Monge García, Zhongping Jian, Jos J. Settels, Charles Hunley, Maurizio Cecconi, Feras Hatib, Michael R. Pinsky

**Affiliations:** 1 Unidad de Cuidados Intensivos, Hospital Universitario SAS de Jerez, C/Circunvalación, s/n, 11407 Jerez de la Frontera, Spain; 2 Edwards Lifesciences, Irvine, CA USA; 3 Orlando Regional Medical Center, Orlando Health, Orlando, FL USA; 4 Department Anaesthesia and Intensive Care Units, Humanitas Research Hospital, Humanitas University, Milan, Italy; 5 Department of Critical Care Medicine, University of Pittsburgh School of Medicine, Pittsburgh, USA

**Keywords:** Ejection fraction, Systolic function, Diastolic function, Contractility, Ventriculo-arterial coupling, Ventricular efficiency, Afterload, Preload, Arterial elastance

## Abstract

**Background:**

The aim of this study was to quantify the impact of different cardiovascular factors on left ventricular ejection fraction (LVEF) and test a novel LVEF calculation considering these factors.

**Results:**

10 pigs were studied. The experimental protocol consisted of sequentially changing afterload, preload and contractility. LV pressure–volume (PV) loops and peripheral arterial pressure were obtained before and after each intervention. LVEF was calculated as stroke volume (SV)/end-diastolic volume (EDV). We studied global cardiac function variables: LV end-systolic elastance (Ees), effective arterial elastance (Ea), end-diastolic volume and heart rate. Diastolic function was evaluated by means of the ventricular relaxation time (*τ*) and ventricular stiffness constant (*β*) obtained from the end-diastolic PV relationship. Ventriculo-arterial coupling (VAC), an index of cardiovascular performance, was calculated as Ea/Ees. LV mechanical efficiency (LVeff) was calculated as the ratio of stroke work to LV pressure–volume area. A linear mixed model was used to determine the impact of cardiac factors (Ees, Ea, EDV and heart rate), VAC and LVeff on LVEF during all experimental conditions. LVEF was mainly related to Ees and Ea. There was a strong relationship between LVEF and both VAC and LVeff (*r*^2^ = 0.69 and *r*^2^ = 0.94, respectively). The relationship between LVEF and Ees was good (*r*^2^ = 0.43). Adjusting LVEF to afterload (${\text{LVEF}}_{\rm EA} = {\text{EF}} \times \sqrt {\text{Ea}}$) performed better for estimating Ees (*r*^2^ = 0.75) and improved the tracking of LV contractility changes, even when a peripheral Ea was used as surrogate (Ea = radial MAP/SV; *r*^2^ = 0.73).

**Conclusions:**

LVEF was mainly affected by contractility and afterload changes and was strongly related to VAC and LVeff. An adjustment to LVEF that considers the impact of afterload provided a better assessment of LV contractility.

## Background

Currently, the most commonly method for the assessment of left ventricular (LV) systolic function in clinical practice is based on estimates of LV ejection fraction (LVEF) by either direct measures of ventricular volumes during cardiac catheterization or using two-dimensional echocardiography. LVEF characterizes LV performance by expressing ventricular ejection (stroke volume, SV) as a fraction of the preload (end-diastolic volume, EDV). LVEF is a strong independent predictor of mortality in patients with both heart failure and acute myocardial infarct [1–3] and plays an essential role in therapeutic decision making [4, 5]. However, LVEF is an inaccurate marker of LV intrinsic contractility [6, 7], because it is strongly influenced by LV loading conditions. For example, LVEF’s ability to detect LV systolic dysfunction has been recently questioned in septic shock patients [8, 9]. A normal LVEF associated with a severely depressed arterial tone, as described in sepsis, can be observed even in the presence of a severely impaired LV intrinsic contractility [9]. Furthermore, changes in LVEF following the introduction of vasoconstrictors have been questioned to reflect actual changes in LV contractility, but rather to unmask prior LV dysfunction when LV afterload has been corrected [9, 10].

Since LVEF must represent the interaction of several cardiovascular factors, we aimed to independently assess the contribution of different determinants affecting LVEF and also to test a novel LVEF calculation for estimating actual LV contractility considering the impact of these determinants. We performed this analysis on hemodynamic data also reported for another study addressing different issues [11]. This study is a continuation of our previous works about central and peripheral hemodynamics [11, 12] and represents a physiological exploration of the determinants of LVEF without any commercial interest.

## Methods

### Animals

The study protocol was approved by the Institutional Animal Care and Use Committee (IACUC) at the Edwards Research Center and performed in accordance with the USDA Animal Welfare Act regulations (AWArs), and the Guide for the Care and Use of Laboratory Animals (ILAR, NAP, Washington, DC, 2010, 8th edition).

Ten anaesthetized and mechanically ventilated adult Yorkshire pigs weighting 81 ± 6 kg were studied. Animals were premedicated with intramuscular telazol (4.4 mg kg^−1^), ketamine (2.2 mg kg^−1^) and xylazine (1.1 mg kg^−1^). They were orally intubated and mechanically ventilated in a volume-controlled mode (FIO_2_ 60–80%, tidal volume 10 ml kg^−1^ at respiratory rate 13–15 cycles min^−1^). Following endotracheal intubation, general anesthesia was maintained with isoflurane 1.5–2.5% and a mixture of oxygen, air and/or nitrous oxide. Fluid maintenance was provided by an intravenous infusion of Ringer’s lactate solution at 2–4 ml kg^−1^ h^−1^. Rectal temperature was monitored and kept between 36 and 37 °C using a heating pad. Animal anesthesia was monitored and recorded approximately every 15 min for the duration of the experimentation.

Instantaneous LV pressure–volume (PV) measurements were obtained from a dual-field conductance catheter and a high-fidelity pressure sensor (CA71083PL, CD Leycom, Zoetermeer, the Netherlands) connected to a PV signal processor (Inca^®^, CD Leycom). The catheter tip was positioned in the LV apex and the correct placement confirmed by fluoroscopy and the examination of the segmental LV PV loops. Radial pressure was continuously recorded with a fluid-filled pressure transducer (FloTracIQ sensor, Edwards Lifesciences, Irvine, CA, USA) using the EV1000 monitor (Edwards Lifesciences).

### Data collection and analysis

Volume signal calibration consisted of the determination of cardiac output (CO) by the standard thermodilution method and correction for parallel conductance by the hypertonic saline method [13, 14]. Calibration was performed before starting the experimental protocol and repeated after the fluid bolus stage. LV pressure–volume signals were recorded at 250 Hz sampling rate, filtered using a 25 Hz low-pass filter and analyzed in a dedicated software (Conduct NT, version 3.18.1, CD Leycom).

*Global cardiac function, ventriculo*-*arterial coupling and LV mechanical efficiency* Before and after each experimental stage, three partial occlusions of the inferior vena cava (IVC) were performed using a Fogarty balloon during apnea. This procedure was repeated if ectopic beats were detected. LV end-systolic elastance (Ees), a load-independent measure of LV contractility [15], was determined as the slope of the end-systolic pressure–volume relationship during the first 10 s of the IVC occlusion, calculated from the linear regression analysis of the maximal elastance points on each cardiac cycle, defined as *E*(*t*) = *P*(*t*)/*V*(*t*) − *V*_0_, where *V*_0_ is volume-axis intercept or the LV unstressed volume [13]. End-systolic pressure (Pes), stroke volume (SV), CO, EDV and end-systolic volume (ESV), end-diastolic pressure, LVEF, effective arterial elastance (Ea = Pes/SV, a lumped parameter of LV afterload) [16] and radial arterial pressure were calculated from 3 to 5 beats in steady-state conditions during apnea just before the IVC occlusion. Ventriculo-arterial coupling (VAC) was computed as the ratio of Ea and Ees [17]. LV pressure–volume area (PVA) represents the total LV mechanical energy and was determined as the sum of stroke work (SW, or the integrated area within each LV PV loop) and potential energy (PE), where PE = Pes × (ESV − *V*_0_)/2. The ratio of SW/PVA, expressed as a percentage, represents therefore the LV mechanical efficiency (LVeff) [18]. As Pes can be estimated from peripheral mean arterial pressure (MAP), a peripheral Ea was also calculated as: Ea_periph_ = radial MAP/SV [12].

*LV diastolic function* We calculated the LV chamber stiffness, a load-independent index of diastolic function representing the passive viscoelastic properties of the LV, from the exponential curve fit of the end-diastolic pressure–volume relationship (EDPVR) during the IVC maneuver, as ${\text{EDP}} = \alpha \times e^{{\beta \times {\text{EDV}}}}$, being α the curve fitting constant and *β* the LV chamber stiffness constant [19]. The time constant of the isovolumetric LV active relaxation (*τ*) was calculated as the time from d*P*/d*t*_min_ until LV pressure reaches half value at the d*P*/d*t*_min_ [19, 20]. End-diastole was defined as the moment on the peak of the R wave on the EKG.

### Experimental protocol

Before starting the protocol, animals received fluid resuscitation (Voluven^®^, 130/0.4, Fresenius Kabi Deutschland GmbH, Bad Homburg, German) until no significant change in CO was observed. Then they were allowed to stabilize for at least 10 min (heart rate and MAP variation < 5%). The study protocol consisted of three consecutive stages with up and down interventions each: changes in afterload (phenylephrine and nitroprusside), preload (bleeding and fluid bolus), and contractility (esmolol and dobutamine). The experiment started with the afterload interventions: Animals were treated with sodium nitroprusside (100–200 mg kg^−1^ min^−1^) to decrease MAP to 40% from baseline (but not below 50 mmHg for allowing an adequate hemodynamic tolerance during the IVC occlusions), followed by recovery to baseline status. Then, they were treated with a phenylephrine infusion (30–120 mg kg^−1^ min^−1^) to increase MAP by 40% mmHg from baseline and were allowed to recover. Subsequently, for preload interventions, the animals were submitted to a stepwise bleeding of 12 ml kg^−1^ (50 ml min^−1^) and the blood stored into a heparinized sterile bag. Then, the blood was reinfused at 50 ml min^−1^, and a fluid bolus of 10 ml kg^−1^ of colloid in 5 min was infused. After the fluid administration, the contractility interventions followed: An esmolol infusion was introduced at 50 µg kg^−1^ min^−1^ and increased until decreasing LV d*P*/d*t*_max_ by 50% from its previous value (maximal dose: 200 µg kg^−1^ min^−1^). Then, the esmolol infusion was stopped and, after a period of recovery, the animals were treated with dobutamine (5 µg kg^−1^ min^−1^) to increase LV d*P*/d*t*_max_ by 50%. LV PV loops and radial arterial pressure were obtained before and after each intervention.

### Statistical analysis

Data are expressed as the mean ± SD, unless otherwise stated. Data normality was checked by the Shapiro–Wilk test. A linear mixed-effects model analysis was used to identify the cardiac variables (fixed effects: Ees, Ea, EDV and heart rate) associated with changes in LVEF. We also analyzed the relationship between LVEF with VAC and LVeff. Models were constructed using individual animals as subjects for random factors, and sequential experimental stages as repeated measurements. A Toeplitz covariance structure was selected based on the corrected Akaike’s information criteria [21, 22]. Model parameters were estimated via the restricted maximum likelihood method and the estimated fixed effects quantified by the estimated value (95% confidence interval). Linear regression analysis was used for determining the relationship between continuous variables. Fisher *z* test was used for comparing correlations. Four-quadrant plots were used for assessing concordance between Ees and different LVEF calculations. Concordance was defined as the percentage of data in which the direction of change agreed. Excellent concordance was assumed when the concordance rate was ≥ 90%. A *p* value < 0.05 was considered statistically significant. All statistical analyses were performed using MedCalc Statistical Software version 18.8 (MedCalc Software bvba, Ostend, Belgium; https://www.medcalc.org; 2016) and SPPS (SPSS 21, SPPS Inc, Chicago, IL).

## Results

*Evolution of main LV variables during different experimental conditions* A detailed description of the main changes in hemodynamics associated with the various interventions was previously reported [11]. Complementary data about changes in VAC, LVeff and diastolic function are reported in Tables 1 and 2.

**Table 1:** Evolution of ventriculo-arterial coupling and left ventricular mechanical efficiency during different experimental stages

	VAC (a.u.)		LV efficiency (%)	
	Before	After	Before	After
Afterload				
Phenylephrine	2.06 ± 0.51	2.36 ± 0.51*	61 ± 9	55 ± 10*
Nitroprusside	2.00 ± 0.49	1.42 ± 0.44^†^	61 ± 10	72 ± 11^‡^
Preload				
Bleeding	2.02 ± 0.61	1.57 ± 0.51*	58 ± 11	67 ± 11^†^
Fluid bolus	1.72 ± 0.63	2.06 ± 0.73*	65 ± 12	57 ± 12*
Contractility				
Esmolol	1.50 ± 0.36	2.43 ± 0.61^†^	68 ± 7	47 ± 3^‡^
Dobutamine	1.71 ± 0.47	1.14 ± 0.34^‡^	65 ± 7	75 ± 1^‡^

Data are presented as mean ± SD

*A.u*. arbitrary units, *VAC* ventriculo-arterial coupling, *LVeff* left ventricular mechanical efficiency

*p < 0.05, ^†^*p* ≤ 0.001 ,^‡^*p* ≤ 0.0001 versus “before” stage

**Table 2:** Evolution of left ventricular diastolic function variables

	*τ* (tau) (ms)		LV stiffness (*β*)	
	Before	After	Before	After
Afterload				
Phenylephrine	29.3 ± 2.9	33.5 ± 3.24^†^	0.013 ± 0.003	0.014 ± 0.003*
Nitroprusside	27.4 ± 3.4	22.4 ± 2.2^‡^	0.013 ± 0.003	0.012 ± 0.003^†^
Preload				
Bleeding	27.5 ± 3.5	22.8 ± 3.2^‡^	0.012 ± 0.003	0.012 ± 0.003
Fluid bolus	25.4 ± 4.3	28.9 ± 4.1^†^	0.012 ± 0.003	0.011 ± 0.002*
Contractility				
Esmolol	27 ± 3.8	35 ± 5.4*	0.013 ± 0.002	0.012 ± 0.003
Dobutamine	27.1 ± 4	24.4 ± 4.5*	0.012 ± 0.002	0.012 ± 0.002

Data are presented as mean ± SD

*τ (tau)* time constant of the isovolumetric LV pressure relaxation, *β* LV chamber stiffness constant obtained from the end-diastolic pressure–volume relationship

*p < 0.05, ^†^*p* ≤ 0.001 ,^‡^*p* ≤ 0.0001 versus before stage

*Impact of global and diastolic function on LVEF* The contribution of different cardiovascular variables on LVEF is detailed in Table 3. The main determinants of LVEF were Ees and Ea, and to a lesser extent EDV and heart rate. There was an inverse relationship between LVEF and Ea; therefore, for a given LV contractility state, a change in Ea will independently change LVEF in a reciprocal fashion. The association of EDV and heart rate to LVEF was minimal when compared with the prominent impact of Ees and Ea. Considering the estimate fixed effects shown in Table 2, the decrease in EDV during bleeding (from 234 + 50 ml to 211 ± 57 ml) would only explain a 3% in the observed LVEF increase after bleeding (from 49 ± 11% to 55 ± 13%), while other factors such as a decreased Ea would also explain the overall LVEF increase. On the other hand, the increase in EDV after fluid administration (from 215 ± 52 ml to 259 ± 47 ml) would explain the 5% decrease observed in LVEF (from 54 ± 13% to 47 ± 10%), while a decrease in Ees and heart rate would also contribute to the net effect in LVEF after volume expansion. Moreover, for a relative 10% increase in Ees, Ea, EDV and heart rate, LVEF will change by 4.5%, − 2.9%, − 0.01% and 0.02%, respectively. Even both active and passive diastolic function variables, *τ* and LV chamber stiffness constant (*β*), had a significant relationship with LVEF, this was small considering the minor changes observed in diastolic function during the study. For example, for a change in LV stiffness constant of 0.001, as seen during afterload interventions or during fluid administration, LVEF will alter by 1.3%.

**Table 3:** Estimated values of different variables on left ventricular ejection fraction (LVEF) according to a linear mixed-effects model analysis

Fixed effects	Estimate	95% confidence interval	*p* value
Global cardiac function			
Ees (mmHg ml^−1^)	44.89	34.28 to 55.49	< 0.001
Ea (mmHg ml^−1^)	− 29.47	− 33.26 to − 25.68	< 0.001
LV EDV (ml)	− 0.12	− 0.15 to − 0.08	< 0.001
Heart rate (bpm)	0.17	0.05 to 0.29	0.006
Diastolic function			
LV *β* constant (a.u.)	1340.3	474.9 to 1932.6	< 0.001
τ (ms)	− 1.28	− 1.49 to − 1.07	< 0.001
VAC (Ea/Ees) (a.u.)	− 10.6	− 11.85 to − 9.37	< 0.001
LV mechanical efficiency (%)	0.87	0.85 to 0.90	< 0.001

Estimate reflects the average change in the LVEF per unit increase of each fixed effect

*Ees* left ventricular end-systolic elastance; *Ea* effective arterial elastance, *LV* left ventricle, *EDV* end-diastolic volume, *EDPVR* slope of the end-diastolic pressure–volume relationship, *β* LV chamber stiffness constant obtained from the end-diastolic pressure-volume relationship, *τ (tau)* time constant of the isovolumetric LV pressure relaxation, *VAC* ventriculo-arterial coupling

**Fig. 1:**
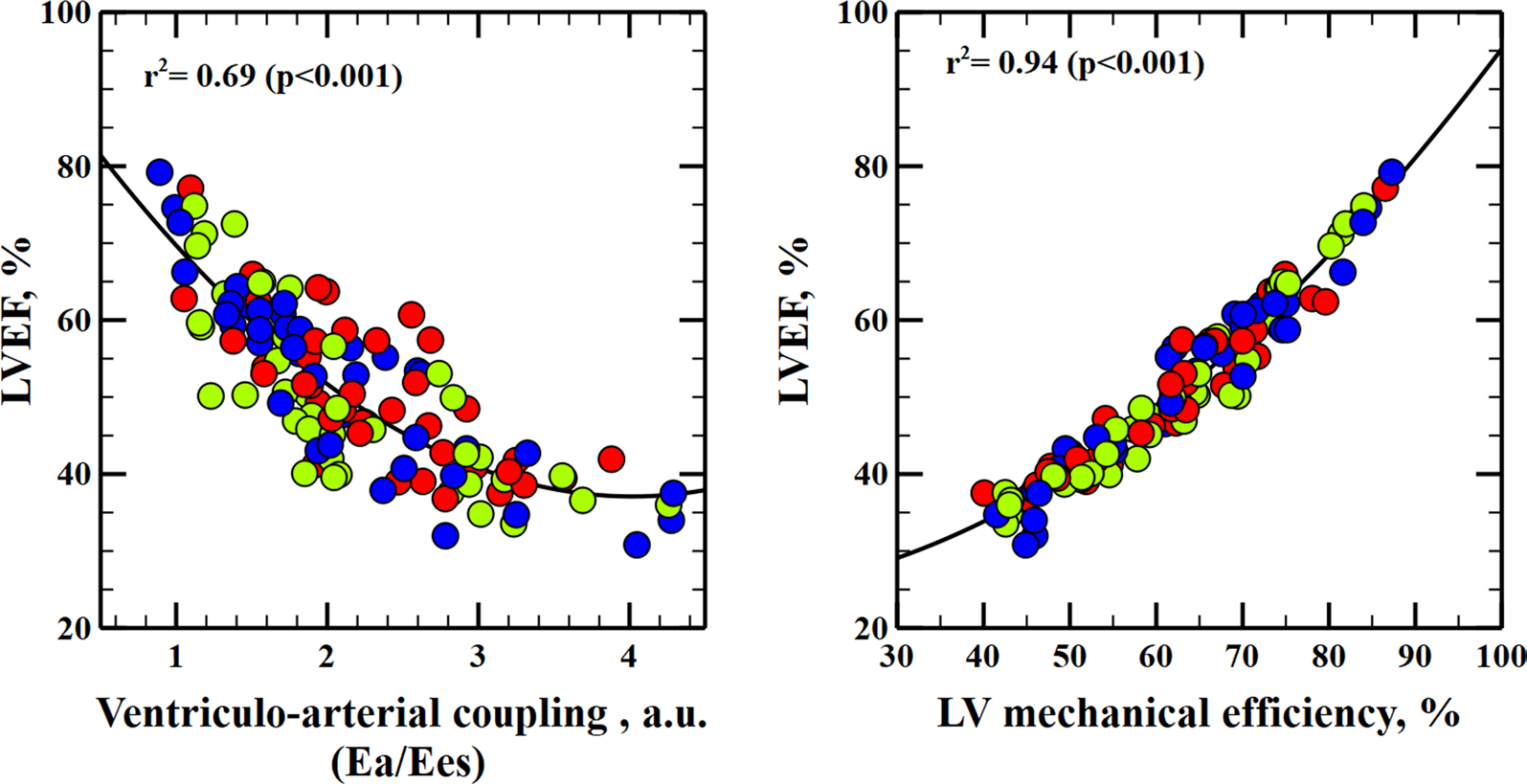
Relationship between ventriculo-arterial coupling, left ventricular mechanical efficiency and left ventricular ejection fraction. Left: linear regression analysis between ventriculo-arterial coupling (VAC), calculated as the ratio between effective arterial elastance (Ea) and left ventricular end-systolic elastance (Ees), and left ventricular ejection fraction (LVEF). Right: linear regression analysis between left ventricular mechanical efficiency, calculated as the ratio between stroke work (SW) and the left ventricular pressure–volume area (PVA), and left ventricular ejection fraction. Colors inside circles represent different experimental interventions: red, afterload; green: preload; blue: contractility

*LVEF as an integrated index of cardiovascular performance* LVEF was strongly related to VAC and LVeff (Table 2 and Fig. 1). A decrease in LVEF was related to a higher VAC and a lower LVeff. A decrease in 10% in LVeff was associated with a parallel 9% reduction in LVEF.

*Performance of an afterload*-*corrected EF for estimating Ees* Given the predominant effect of Ea, we proposed an afterload-adjusted LVEF using a simple nonlinear approach for estimating LVEF:

$${\text{LVEF}}_{\rm EA} = {\text{EF}} \times \sqrt {\text{Ea}} ,$$

which it can be simplified as:

$${\text{LVEF}}_{\rm EA} = \frac{\text{SV}}{\hbox{EDV}} \times \sqrt {\frac{\text{Pes}}{\hbox{SV}}} .$$

Therefore,

$${\text{LVEF}}_{\rm EA} = \sqrt {\left( {{\text{Pes}} \times {\text{SV}}} \right)} /{\text{EDV}}.$$

**Fig. 2:**
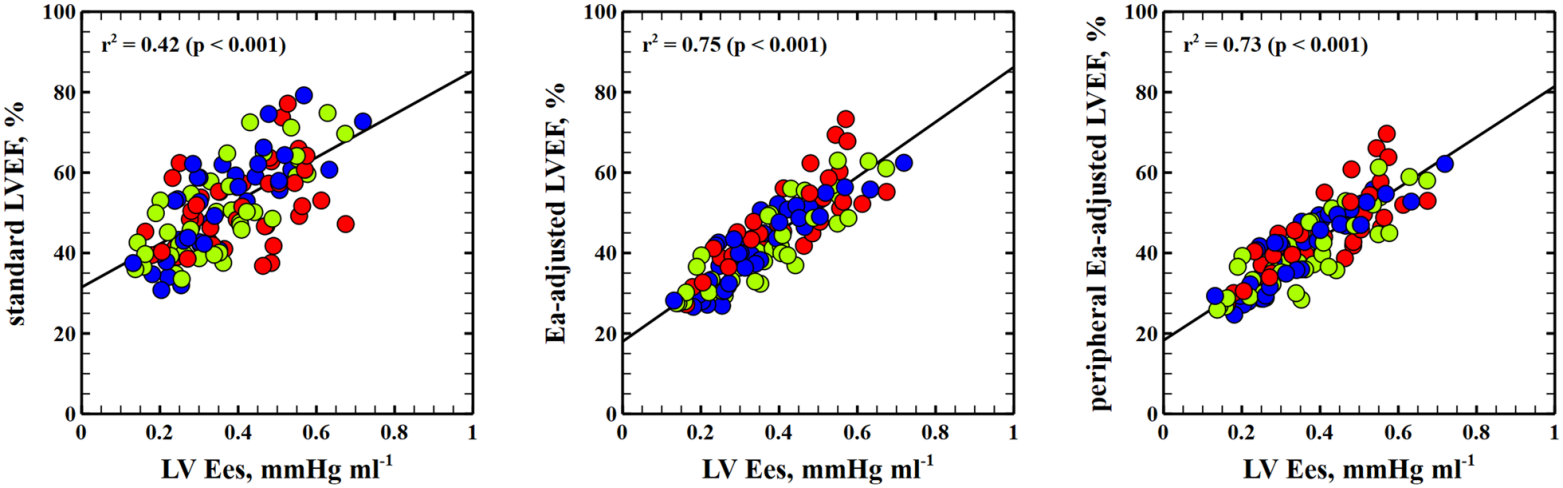
Relationship between left ventricular end-systolic elastance (Ees) and standard left ventricular ejection fraction (LVEF) calculation, Ea-adjusted LVEF and peripheral Ea-adjusted LVEF. Linear regression analysis for left ventricular end-systolic elastance (Ees) and standard LVEF, LVEF corrected to effective arterial elastance (Ea = left ventricular end-systolic pressure/left ventricular stroke volume) and peripheral Ea-adjusted LVEF (Ea_periph_ = radial mean arterial pressure/stroke volume). Colors inside circles represent different interventions: red, afterload; green: preload; blue: contractility

Since LVEF mainly depends on LV contractility and afterload, this modification of the standard formula would consider both the direct relationship of LVEF with Ees and the inverse correlation with Ea. By using the square root function of Ea, it would also take into account that the impact of afterload is greater at low levels of contractility [6]. Although we tested different potential linear and nonlinear approaches, we selected this adjustment based on the highest correlation with Ees (*r*^2^ = 0.75 vs *r*^2^ = 0.43 for standard LVEF calculation; *p* < 0.0001 for the comparison, Fig. 2), its simplicity and its clinical feasibility. This improvement still held even when Ea was estimated using radial MAP as surrogate for Pes (${\text{peripheral LVEF}}_{\rm EA} = \sqrt {\left( {{\text{radial MAP}} \times {\text{SV}}} \right)} /{\text{EDV}}$): *r*^2^ = 0.73 (*p* = 0.0001 vs standard LVEF calculation). The combination of an afterload and preload adjustment did not improve this relationship (*r*^2^ = 0.73; *p* = 0.6635 vs LVEF_EA_). A simple approach that considered a linear relationship between Ea and LVEF (${\text{LVEF}}_{\rm EA linear} = {\text{EF}} \times {\text{Ea}}$) also did not improve the relationship with Ees (*r*^2^ = 0.61; *p* = 0.0424 vs LVEF_EA_).

**Fig. 3:**
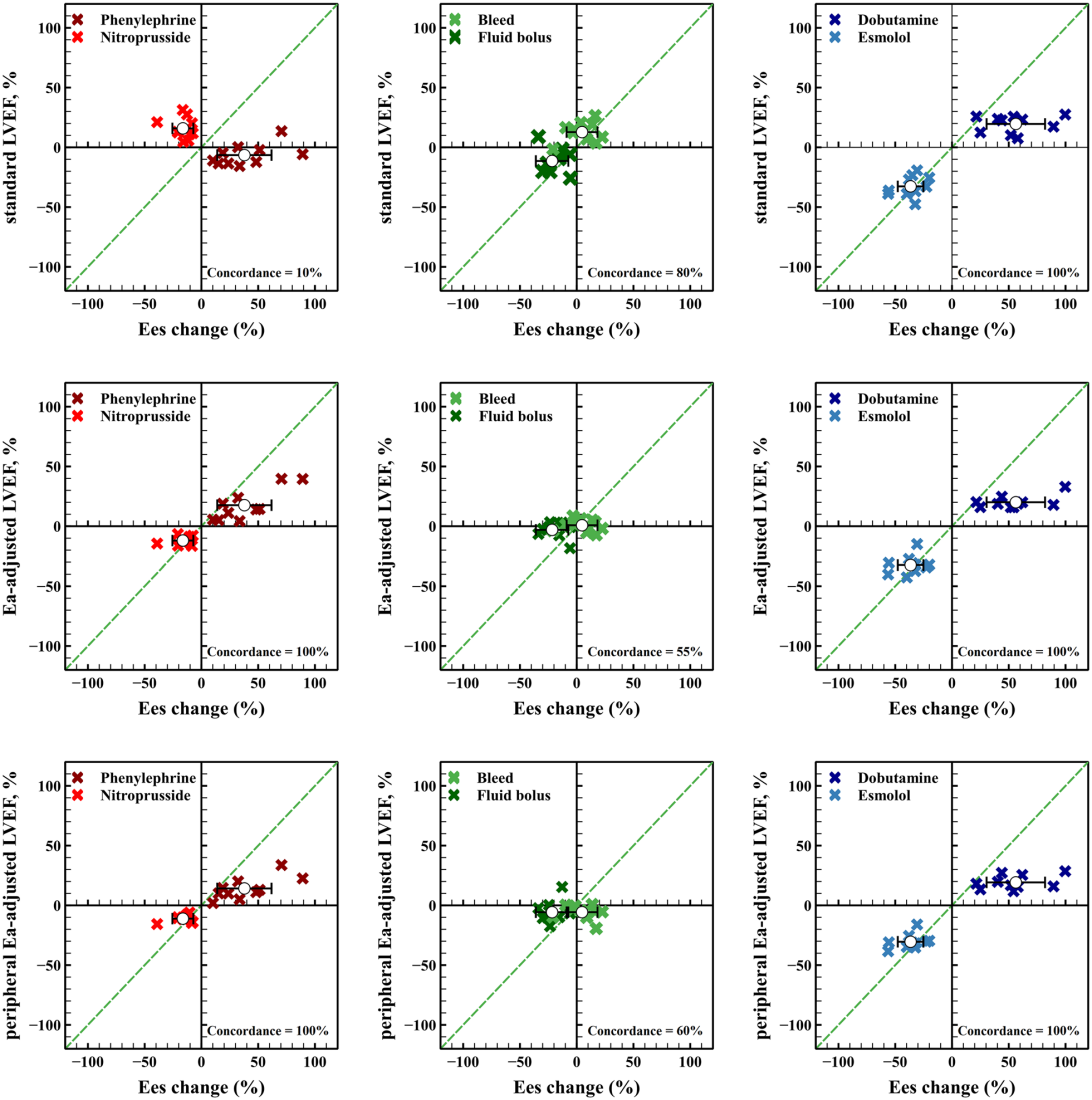
Concordance analysis for percentage changes in different approaches for estimating left ventricular contractility and end-systolic elastance (Ees) during different experimental stages. Four-quadrant plots showing the relationship between percentage changes in left ventricular (LV) end-systolic elastance (Ees) and different approaches studied: standard LVEF calculation, LVEF adjusted to effective arterial elastance (Ea = left ventricular end-systolic pressure/stroke volume) and peripheral Ea-adjusted LVEF (Ea_periph_ = radial mean arterial pressure/stroke volume). Excellent trending capability was assumed when ≥ 90% of the data lie in the right-upper and the left-lower quadrants. Open circles with bars represent the mean percentage change on each stage. Dashed green lines represent the line of equality

*Performance comparison for tracking changes in Ees during different experimental conditions* Concordance analysis for different approaches for tracking Ees changes is shown in Fig. 3. While all three methods showed perfect tracking of Ees during contractility changes, when afterload was changed, standard LVEF poorly reflected Ees variations. On the other hand, both central and peripheral Ea-adjusted LVEF significantly improved the tracking of Ees during afterload interventions, while standard LVEF was better for tracking Ees changes during preload variations, although these changes were significantly smaller in amplitude compared to those observed during contractile and afterload variations.

## Discussion

This study confirms that LV Ees and Ea are the main determinants of LVEF. Not surprisingly, we also corroborated the strong relationship between LVEF and both VAC and LVeff, which are mathematically coupled to Ees and Ea. Thus, LVEF is mainly determined by the coupling between the LV systolic function and the arterial system and less so to changes in preload and heart rate. As such, LVEF represents more of a global index of cardiovascular performance rather than an absolute measure of LV contractility. We also propose an afterload correction to LVEF that significantly improved the performance over the standard LVEF calculation for estimating LV contractility during changes in loading conditions, while based on easily available parameters.

Ideally, an index of contractility index should be sensitive to changes in myocardial contractile state but indifferent to loading conditions [6]. Although LVEF has been traditionally used as a clinical measure of LV systolic performance, its dependence on preload and especially afterload changes have been well documented experimentally over five decades ago by Krayenbuhl et al. [23], later corroborated in isolated canine ventricles by Kass et al. [24] and theoretically described by Robotham [6]. However, the recognition that LVEF does not only depend solely upon myocardial contractility but also upon other determinants of the ventricular function has been increasingly recognized in critically ill patients with the growing use of echocardiography in ICU to assess LV function [3, 9, 25]. For example, the afterload influence in LVEF has been particularly evident and documented in septic patients [8, 9, 26]. Impaired intrinsic contractility and vasoplegia are the hallmark of the hemodynamic disorders in septic shock [27, 28]. Under these conditions, a normal LVEF may not necessarily indicate a normal LV contractility, but rather reflect the profoundly impaired LV afterload [9, 29]. Similarly, a low LVEF within the context of a decreased LV afterload would support the diagnosis of severely depressed LV systolic function. On the other hand, a reduction in LVEF after correcting arterial hypotension with vasopressors need not reflect a worsening of LV systolic function, but only the unmasking of existing LV dysfunction by normalization of LV afterload [10]. Only if LVEF still remains low after restoring arterial pressure and afterload, then an impairment in LV contractility is likely. This reasoning could explain the large variability of LV dysfunction reported in previous clinical studies and the lack of association between LVEF and mortality in septic shock [8, 30]. As loading conditions can be significantly altered by vasopressors or fluid administration, differences on the timing and the amount of hemodynamic resuscitation could have influence the reported value of LVEF regardless the intrinsic contractile state. Accordingly, considering the multiple factors that can influence LVEF in septic shock patients, where perturbations of loading conditions are particularly frequent, the interpretation of LVEF as an index of LV contractility should be done cautiously and after considering the potential impact of these factors [25].

While afterload changes largely affected LVEF, preload variations, even when abrupt and of large amplitude, such as during bleeding and fluid loading, failed to greatly influence LVEF. This was particularly evident even if CO significantly increased during fluid loading (from 7.89 ± 1.7 l/min to 9.11 ± 2.42 l/min; *p* = 0.01), while LVEF showed only a modest decrease (from 54 ± 13% to 47 ± 10%; *p* = 0.02). This is presumably related to the fact that LVEF normalized changes in SV by EDV, while a reduction in LVEF after fluid infusion would be associated with an increase with EDV without a subsequent improvement in SV (non-preload dependency). Therefore, a decrease in LVEF after fluid administration could be indicative of a lack of preload responsiveness, while an unchanged or an increased LVEF would denote a significant increase in SV with volume administration (fluid responsiveness).

Additionally, LVEF is a function of both SV and EDV. Patients with sustained lusitropy but preserved SV will paradoxically have a reduced LVEF, owing to the increased EDV, but have better cardiovascular reserve. This assumption supports the multiple studies reporting a decreased LVEF in survivors of severe sepsis as compared to non-survivors [31]. Non-survivors presumably are unable to relax appropriately during diastole, while both survivors and non-survivors have sepsis-induced impaired systolic function. This hypothesis is supported by the association found between diastolic disfunction and mortality in septic patients [32, 33]. As we did not also study the impact of sepsis in our porcine model, we cannot speculate further on this hypothesis. Moreover, the effects of our interventions on diastolic function, though measurable, were small and not relevant, because they minimally altered EDV. However, in the setting of altered diastolic function, as may occur in sepsis and LV hypertrophy, these effects may become relevant. Further focused investigation of these issues would be needed to address these concerns.

Our in vivo experimental study with an intact cardiovascular system confirms previous observations about the influence of the cardiac loading conditions on LVEF [23, 24, 34–36], but more importantly, it also provides the empirical demonstration about the real nature of LVEF. Since LVEF is primarily governed by the influence of Ees and Ea, it mainly reflects the balance between LV contractile function and the arterial system. Therefore, LVEF should be considered as a ventriculo-arterial coupling index and a variable mostly related to LV systolic mechanical efficiency, but not a pure measure of LV systolic performance [6, 37]. Furthermore, even if LVEF is associated with a unique VAC and LV mechanical efficiency state, the same LVEF level can be obtained with different values of Ees and Ea. Thus, the clinical utility of measures of LVEF under conditions of varying cardiovascular states is unclear and may be misleading.

Thirty years ago Robotham stated that LVEF “reflects the integrated system’s ability to cope, under the conditions at the time of measurement, with abnormalities in preload, afterload and/or contractility”, and therefore, “ejection fraction becomes a measure, not of ventricular performance, but of the integrated system’s performance in dealing with a pathological process” [6]. Although his measures were less accurate than ours, our data agree with his fundamental observation. Accordingly, an abnormal LVEF represents the inability of the cardiovascular system to modulate the current contractile and loading conditions for sustaining normal homeostasis. On the contrary, a normal LVEF does not necessarily mean that LV contractility is preserved, but it rather represents a composite variable expressing the cardiovascular system’s response to the current loading and contractile conditions. Practically speaking, a low LVEF should always be considered as a sign of impaired cardiovascular function, reflecting an unfavorable LVeff and VAC, but it does not inform about the underlying mechanisms leading to its low value, which could be an impaired contractility, increased afterload or both. Furthermore, a normal LVEF, particularly in critically ill patients, should never be interpreted as evidence of normal LV systolic function, especially in the presence of hypotension and systemic hypoperfusion. Relevant to this last point, increased LVEF due to a predominant reduction on LV afterload, even in the presence of an impaired LV contractility, has been associated with a higher mortality [8, 38, 39].

While standard LVEF remains of clinical interest, since it gives a global picture of LV pump function for the given contractility and loading conditions, we propose reporting an Ea-adjusted LVEF to significantly improve the ability of LVEF to tracking changes in Ees during acute perturbations of LV afterload, without affecting the performance during changes in contractility. Moreover, we have recently demonstrated that radial, femoral and aortic MAP values are interchangeably as valid surrogates for the estimation of Pes, so LVEF can be adjusted using a peripheral estimation of Ea regardless the arterial pressure measurement site [12]. Since the additional information required to calculate LVEF_EA_ can be obtained using currently available measures of arterial pressure and ventricular function, its application for estimating LV contractility could be easily implemented. This afterload-corrected LVEF could be particularly helpful for identifying those patients with depressed LV contractility in the setting of altered loading conditions, as seen during septic shock. Furthermore, identification of depressed contractility by the Ea-corrected LVEF in the context of a persistent tissue hypoperfusion could theoretically better define the population potentially eligible for inotropic therapy. However, the usefulness and superiority of these surrogates in the clinical practice needs further clinical validation.

Our results require a few comments. First, we did not use echocardiography for calculating LVEF, which is the standard in clinical practice. Instead, we used the data obtained from the LV PV analysis by the classical conductance catheter technique. This method has been demonstrated to provide the most comprehensive description of the LV function [40], whereas estimation of LVEF by standard echocardiography relies on geometrical assumptions and is prone to technical inaccuracies and inter-/intra-observer variability. Thus, echocardiographic estimates of LVEF should show even greater variance than our findings, underscoring further the lack of sensitivity of LVEF estimates to track LV contractility. However, our results are not limited to the use of this conductance catheter methodology, since they should remain valid to any estimation of LVEF, such as that obtained from the 3D echocardiography or global ejection fraction from transpulmonary thermodilution. However, as our LVEF estimation was based on the volumetric data from the conductance catheter, these clinical estimates of LVEF would still rely on the accuracy of their measurements and their assumptions about the LV geometry. Finally, our study was performed on pigs receiving anesthesia, so our results should be interpreted with caution when extrapolating to human cardiovascular physiology in awake individuals.

## Conclusions

Changes in LVEF are mainly influenced by LV contractility and afterload and strongly related with ventriculo-arterial coupling and LV mechanical efficiency. Therefore, LVEF primarily represents an integrated index of cardiovascular performance and LV mechanical efficiency rather than an actual measure of LV contractility. We proposed an afterload-adjusted LVEF, which significantly improved the ability to track LV contractility.

## Abbreviations

LV: left ventricle
Ees: end-systolic elastance
LVeff: LV mechanical efficiency
LVEF: left ventricular ejection fraction
LVEF_Ea_: left ventricular ejection fraction adjusted to effective arterial elastance
PV: pressure–volume
CO: cardiac output
IVC: inferior vena cava
Pes: left ventricular end-systolic pressure
SV: stroke volume
EDV: left ventricular end-diastolic volume
ESV: left ventricular end-systolic volume
Ea: effective arterial elastance
*E*: elastance
*V*_0_: left ventricular unstressed volume
MAP: mean arterial pressure
AICc: Akaike’s information criteria
ICU: intensive care unit
